# Liver Dysfunction Associated with Intravenous Methylprednisolone Pulse Therapy in Patients with Graves' Orbitopathy

**DOI:** 10.1155/2015/835979

**Published:** 2015-06-28

**Authors:** Hiroyuki Eguchi, Junichi Tani, Saori Hirao, Munehisa Tsuruta, Ichiro Tokubuchi, Kentaro Yamada, Masataka Kasaoka, Yasuo Teshima, Tatsuyuki Kakuma, Yuji Hiromatsu

**Affiliations:** ^1^Division of Endocrinology and Metabolism, Department of Medicine, Kurume University School of Medicine, Kurume 830-0011, Japan; ^2^Department of Ophthalmology, Kurume University School of Medicine, Kurume 830-0011, Japan; ^3^Division of Biostatistics Center, Kurume University, Kurume 830-0011, Japan

## Abstract

Intravenous methylprednisolone (IVMP) pulse therapy is the first-line treatment for the active phase of moderate to severe Graves' orbitopathy (GO). However, acute and severe liver damage has been reported during and after IVMP therapy. In this retrospective study, we investigated risk factors for liver dysfunction during and after IVMP therapy based on 175 Japanese patients with moderate to severe GO and treated at our center between 2003 and 2011. The results showed that seven patients developed severe liver dysfunction with elevated serum alanine aminotransferase (ALT > 300 U/L). Mild (40–100 U/L) and moderate (100–300 U/L) increases of ALT occurred in 62 patients (35%) and 10 patients (6%), respectively. Liver dysfunction was more frequently observed in males, in patients receiving high-dose methylprednisolone, and patients aged over 50 years. Preexistent viral hepatitis was significantly associated with liver dysfunction (65% in patients positive for hepatitis B core antibody and patients positive for hepatitis C virus antibodies). Our study confirmed the association of liver dysfunction with IVMP during and after treatment. It suggests that, in patients with GO, evaluation of preexisting risk factors—including viral hepatitis—and careful weekly monitoring of liver function during IVMP therapy and monthly thereafter for 12 months are warranted.

## 1. Introduction

Intravenous methylprednisolone (IVMP) pulse therapy is the first-line treatment for patients with active-phase moderate to severe Graves' orbitopathy (GO) [1]. IVMP is widely used because it is more effective and better tolerated than oral steroids [2, 3]. However, acute and severe liver damage has been reported after pulse therapy, with a roughly estimated morbidity and mortality of 0.8% and 0.3%, respectively [4]. The cumulative dose of IVMP in four patients with fatal liver failure was 8.3–15 g [4, 5] but slightly higher in three patients who died (10.8 ± 3.6 g) than in four patients who recovered (7.9 ± 2.9 g) [4]. Therefore, the European Group of Graves' Orbitopathy (EUGOGO) now recommends that the cumulative dose of MP should be less than 8 g [1, 6].

The causes of IVMP-associated liver damage are incompletely understood. Thus, the aim of the present study was to investigate the risk factors for liver dysfunction during and after IVMP pulse therapy for GO.

## 2. Materials and Methods

### 2.1. Study Population

This was a retrospective study of 175 Japanese patients with moderate to severe GO who were treated in one center from 2003 to 2013. The mean age of the 118 females and 57 males was 51.7 ± 15.5 years. They had been admitted to our university hospital for GO and were treated with an intravenous injection of 1 g of MP daily for 3 consecutive days per week, repeated for three to six cycles, and followed by a tapering dose of oral prednisolone (20 mg/day for 4 weeks, 15 mg/day for 2 weeks, 10 mg/day for 2 weeks, 5 mg/day for 2 weeks, and 5 mg/2 days for 2 weeks). The daily dose of MP was reduced to 0.5 g except in cases with optic neuropathy after the recommendation by EUGOGO in 2008 [1]. Heart rate and ECG were monitored during the intravenous infusion of MP, administered every 2-3 h. In addition, 100 of the 175 patients were treated with orbital irradiation therapy (2 Gy/day, 10 times; total dose = 20 Gy) either during or after IVMP pulse therapy. All patients were given artificial tear drops to protect the cornea. Histamine receptor 2 antagonists or proton pump inhibitors were administered for all the cases. Bisphosphonates were administered in 82 patients to protect steroid-induced osteoporosis.

### 2.2. Biochemical Examination and Diagnosis of Thyroid Diseases

Thyroid diseases were diagnosed by measuring serum-free triiodothyronine (FT3), free thyroxine (FT4), thyroid-stimulating hormone (TSH), thyroglobulin, anti-thyroglobulin antibody, anti-thyroid peroxidase antibody, and anti-thyrotropin receptor antibodies (TRAbs). TRAbs were measured using three commercial kits: TRAb 1st generation (TRAb Cosmic III, Cosmic, Tokyo, Japan), TRAb 2nd generation, human TRAb (Yamasa, Tokyo, Japan) and TSAb (Yamasa TSAb kit), and thyroid ^123^I uptake on ^123^I scintigraphy. Orbitopathy was estimated by ophthalmologists using a modified NOSPECS classification [7] and the clinical activity score (CAS) [1]. Magnetic resonance imaging was also performed before and after pulse therapy, as previously reported [8]. Graves' disease was detected in 139 patients, 29 patients were euthyroid without a history of Graves' disease, and 7 patients had hypothyroidism without a history of Graves' disease. Orbitopathy with NOSPECS class VI was determined in 8 patients, class V in 3 patients, class IV in 139 patients, class III in 23 patients, and class II in 2 patients.

Liver function tests were performed once a week during pulse therapy and repeated at every visit thereafter for 1 year. Hepatitis B surface antigen (HBsAg), hepatitis B surface antibody (HBsAb), hepatitis B core antibody (HBcAb), and hepatitis C virus antibody (HCVAb) were measured before pulse therapy. The one patient who was HBsAg-positive consulted with a hepatologist, who prescribed 0.5 mg of entecavir, during and after pulse therapy. In addition, 43 patients were HBcAb-positive and 17 were HCVAb-positive. They likewise consulted with hepatologists before pulse therapy. Serum HBV-DNA was not detected in any patient. HBV-DNA and HCV-RNA were also monitored. Liver dysfunction was classified based on serum alanine aminotransferase (ALT) and total bilirubin levels as mild (ALT: 40–100 U/L), moderate (ALT: 100–300 U/L), or severe (ALT > 300 U/L or total bilirubin: >3 mg/dL).

### 2.3. Clinical Characteristics of Patients with GO

Female and male GO patients significantly differed with respect to age, body mass index (BMI), smoking habits, alcohol habits, and HBcAb positivity before pulse therapy (Table 1). Human TRAb and TSAb levels were significantly higher in female than in male patients before IVMP pulse therapy.

### 2.4. Statistical Analysis

Statistical analysis was performed using JMP Pro software (version 11.0.0, SAS Institute, USA). Data are expressed as the mean ± standard deviation. Statistical comparisons were performed using Student's *t*-test, one-way ANOVA, or Mann-Whitney *U* test for the analysis of continuous variables. The *χ* ^2^ test or Fisher's exact probability test was used to analyze 2 × 2 or 2 × 4 tables. Multivariate logistic regression analyses were carried out to evaluate the risk factors for liver dysfunction, using exact method (LogXact, Cytel Inc., USA). In all tests, a *P* value < 0.05 was considered to indicate significance.

## 3. Results

### 3.1. Liver Dysfunction

Increases of ALT during and/or after pulse therapy were detected in 79 patients (45%) (Table 2). Mild (ALT 40–100 U/L), moderate (ALT 100–300 U/L), and severe (ALT > 300 U/L) increases of serum ALT were measured in 62 patients (35%), 10 patients (6%), and 7 patients (4%), respectively. All patients with severe liver dysfunction were female and most of them were female. Two were HBcAb positive and one of them developed jaundice with ALT 945 U/L, one day after the cessation of IVMP. Her total bilirubin 8 weeks after the cessation of IVMP was 18.45 mg/dL. Single and cumulative doses of IVMP were 0.5 g and 3.5 g, respectively. HBV-DNA was not detected in patients with severe liver dysfunction. Anti-nuclear and anti-single-stranded DNA antibodies were also negative, as were anti-smooth muscle antibody, and anti-double-stranded DNA antibody in those patients. The HBV carrier taking entecavir prescribed by the hepatologist did not show the elevation of ALT.

### 3.2. Factors Associated with Liver Dysfunction during or after Pulse Therapy for GO

Liver dysfunction occurred more frequently in male patients (*P* < 0.0012) and in patients over the age of 50 years (*P* < 0.0009). BMI was significantly higher in patients with mild liver dysfunction than in those without liver dysfunction (23.3 ± 4.33 kg/m^2^ versus 22.0 ± 3.39 kg/m^2^, Student's *t*-test, *P* = 0.043, data not shown). Liver dysfunction was not associated with smoking or alcohol habit but it was associated with a high dose of MP (cumulative dose >8 g versus <8 g, 2 × 2 table, *χ* ^2^ = 6.280, *P* = 0.0122). Preexistent viral hepatitis was significantly associated with liver dysfunction during and after pulse therapy (*P* = 0.0035). HBcAb was positive in 43 GO patients (25%) before pulse therapy. In 28 of them (65%), ALT was significantly increased (*P* = 0.01125), although in most the increase was mild. HCVAb was positive in 17 GO patients (10%) before pulse therapy. In this group, 11 patients (65%) had increased ALT levels (*P* = 0.01132).

### 3.3. Multivariate Logistic Regression Analysis

Multivariate logistic regression analysis showed that age, gender, and cumulative MP dose (>8 g) were associated with liver dysfunction (Table 2).

## 4. Discussion

Although IVMP pulse therapy is widely used as the first-line treatment for active moderate-to-severe orbitopathy, severe related side effects have been reported, the most common of which is hepatotoxicity. In the recent review by Zang et al. [9], the morbidity and mortality of GO patients treated with IVMP pulse therapy were 6.5% and 0.6%, respectively. Fatal hepatotoxicity was reported to be associated with a cumulative dose of IVMP > 8 g. In two studies, the cumulative doses were 8.3–15 g [4, 5]. EUGOGO now recommends that the cumulative dose of IVMP does not exceed 8 g [1].

In our series of 175 patients, seven patients (4.0%) developed severe liver dysfunction. The rate of morbidity was similar to the previous report [9]. The cumulative doses of MP were more than 8 g in 5 out of seven patients. However, single and cumulative doses of IVMP in a patient with jaundice were 0.5 g and 3.5 g, respectively.

Koga et al. [10] reported two fatal cases of HBV carriers after corticosteroid therapy, and the frequent reactivation of HBV after immune suppressive therapy, such as with rituximab, was noted [11]. Therefore, in GO patients during IVMP therapy, the reactivation of HBV leading to acute liver failure remains a concern, although its occurrence is rare [4, 9, 12, 13]. Indeed, in the series of Le Moli et al. [12], none of the 27 patients with GO suffered serious liver damage. Wichary and Gasińska [13] concluded that the risk of HBV reactivation is low, based on their experience with 30 patients treated with IVMP. Those studies suggest that it is difficult to predict who will develop severe liver failure, such that it is important to carefully monitor patients during and after IVMP therapy.

Our study identified risk factors for mild to moderate liver dysfunction during and after IVMP therapy for GO. Among male patients, a mild elevation of ALT was associated with a cumulative dose of IVMP > 8 g; in female patients, a moderate elevation of ALT was associated with age over 50 years. A history of HBV and HCV infection also contributed to a high prevalence of hepatotoxicity, as approximately 25% of our GO patients were HBcAb-positive and 10% were HCVAb-positive before pulse therapy. Within this group, 65% had increased ALT levels during and/or after pulse therapy. Multivariate logistic regression analysis showed that gender, age, and cumulative dose of MP were associated with liver dysfunction. Our study in Japanese patients suggests that viral hepatitis, gender, age, and cumulative dose are predisposing risk factors for hepatotoxicity during and after IVMP therapy. The current study also supports recommendations of a cumulative dose of MP < 8 g. However, as even this dose may not be completely safe, careful monitoring of GO patients receiving IVMP is recommended both during and 12 months after therapy.

Although the mechanisms of mild to severe hepatotoxicity remain unclear, reactivation of viral hepatitis [4, 10, 11], a direct toxicity of MP [12–14], and exacerbation of autoimmune hepatitis have been suggested [15, 16]. Le Moli et al. [12] reported that mild elevations in liver enzymes following IVMP were dose dependent. The toxic effect of glucocorticoids on hepatocytes, leading to drug-induced steatohepatitis, is thought to involve mitochondrial injury because of the impaired *β*-oxidation of fatty acids, with subsequent generation of reactive oxygen species and ATP depletion [17].

Salvi et al. [15] and Marinò et al. [16] reported the exacerbation of autoimmune hepatitis with severe liver dysfunction during IVMP therapy. In our series, two patients were positive for antinuclear and anti-smooth muscle antibodies but in both cases liver dysfunction was mild.

The drug-drug interaction may be another possible mechanism of liver dysfunction [18]. None of patients received aspirin in combination of Ramipril or clopidogrel.

There were several limitations to this study. First, it was retrospective in design. However, it allowed us to assess the effect of single and cumulative doses of IVMP, because in line with the EUGOGO's recommendation we reduced the single dose of IVMP from 1 g to 0.5 g. Another limitation of the study was the small number of patients, which prevented definite conclusions because of the low incidence of severe liver dysfunction. Additionally, no histopathological examinations were done and the effectiveness of IVMP for GO was not evaluated. Therefore, further prospective studies are indicated to assess the hepatotoxicity of IVMP during and after pulse therapy for GO.

In conclusion, liver dysfunction is frequently associated with pulse therapy for GO, both during and after treatment. Our study supports the careful evaluation of preexisting risk factors (especially viral hepatitis, age, gender, body mass index, and smoking history) before initiating IVMP therapy in GO patients. In these patients, strict monitoring of liver function once a week during pulse therapy and every month thereafter for the next 12 months is warranted.

## Figures and Tables

**Table 1:** Clinical characteristic of patients with Graves' orbitopathy.

	Total	Male	Female	Male versus female
	*N* = 175	*N* = 57	*N* = 118	*P* value

Age (yr)	51.7 ± 15.5	55.9 ± 16.3	49.6 ± 11.9	0.012

BMI (kg/m^2^)	22.5 ± 3.7	23.4 ± 3.8	22.1 ± 2.7	0.021

HBcAb (+)	43 (25%)	21 (37%)	22 (19%)	0.0102

HCVAb (+)	17 (10%)	9 (16%)	8 (7%)	0.0677

HBcAb (−) HCVAb (−)	122 (70%)	31 (54%)	91 (77%)	—

IVMP > 8 g	118 (67%)	40 (70%)	78 (66%)	0.5884

Smoking (+)	57 (33%)	27 (47%)	30 (25%)	0.0041

Alcohol (+)	48 (27%)	28 (49%)	20 (17%)	0.0001

CAS	3.1 ± 1.7	3.2 ± 1.8	3.0 ± 1.7	0.8263

TRAb (%)	29.3 ± 27.3	25.8 ± 26.1	30.8 ± 27.8	0.2987

hTRAb (IU/L)	17.7 ± 44.4	8.10 ± 11.4	21.9 ± 52.2	0.0407

TSAb (%)	1262 ± 1480	931 ± 1229	1404 ± 1559	0.0346

				Mean ± SD

BMI, body mass index; HBcAb, anti-hepatitis B core antibody; HCVAb, anti-hepatitis C virus antibody; IVMP, intravenous injection of methylprednisolone; CAS, clinical activity score; TRAb, anti-thyrotrophin antibody; hTRAb, human TRAb; TSAb, thyroid stimulating antibody.

**Table 2:** Risk factors associated with liver dysfunction during and/or after intravenous methylprednisolone pulse therapy for Graves' orbitopathy.

	Number of patients	Liver dysfunction				Univariate	Multivariate analysis		
		ALT (IU/L)				Fisher's exact probability test	Multinomial logit model for an unordered response		
		<40	40–100	100–300	>300		95% CI		2 sided
						2 × 4	Lower	Upper	*P* value
Total	175	96 (55%)	62 (35%)	10 (6%)	7 (4%)				

Male	57	21 (37%)	31 (54%)	5 (9%)	0 (0%)	*χ* ^2^ = 18.34			
Female	118	75 (64%)	31 (26%)	5 (4%)	7 (6%)	*P* = 0.000278	0.1348	1.646	0.01895

HBcA b (+)	43	15 (35%)	24 (56%)	2 (5%)	2 (5%)	*χ* ^2^ = 11.01			
						*P* = 0.01125			
HCVAb (+)	17	6 (35%)	6 (35%)	4 (24%)	1 (6%)	*χ* ^2^ = 11.94			
						*P* = 0.01132			
HBcAb (+) and/or HCVAb (+)	53	19 (36%)	26 (49%)	5 (9%)	3 (6%)	*χ* ^2^ = 11.36 *P* = 0.008867^*∗*^	−0.245	1.417	0.1846
HBcAb (−) HCVAb (−)	122	77 (63%)	36 (30%)	5 (4%)	4 (3%)	—			

Age									
>50 yr	95	41 (43%)	45 (47%)	8 (8%)	1 (1%)	*χ* ^2^ = 20.72	0.03396	1.553	0.03972
≦50 yr	80	55 (69%)	17 (21%)	2 (3%)	6 (8%)	*P* = 0.00004			

IVMP									
>8 g	118	57 (48%)	47 (40%)	9 (8%)	5 (4%)	*χ* ^2^ = 7.187	0.1012	1.640	0.02426
<8 g	57	39 (89%)	15 (26%)	1 (2%)	2 (4%)	*P* = 0.06052			

Smoking									
(+)	57	26 (46%)	24 (42%)	4 (7%)	3 (5%)	*χ* ^2^ = 2.969			
(−)	118	70 (59%)	38 (32%)	6 (5%)	4 (3%)	*P* = 0.3887			

Alcohol									
(+)	48	24 (50%)	19 (40%)	3 (6%)	2 (4%)	*χ* ^2^ = 0.6445			
(−)	127	72 (57%)	43 (34%)	7 (6%)	5 (4%)	*P* = 0.9176			

^*∗*^Compared to patients without HBcAb or HCVAb.

ALT, alanine aminotransferase; HBcAb, anti-hepatitis B core antibody; HCVAb, anti-hepatitis C virus antibody; IVMP, intravenous injection of methylprednisolone.
